# Respiratory Syncytial Virus: Knowledge, Attitudes and Beliefs of General Practitioners from North-Eastern Italy (2021)

**DOI:** 10.3390/pediatric14020021

**Published:** 2022-03-24

**Authors:** Matteo Riccò, Pietro Ferraro, Simona Peruzzi, Alessandro Zaniboni, Silvia Ranzieri

**Affiliations:** 1 AUSL–IRCCS di Reggio Emilia, Servizio di Prevenzione e Sicurezza Negli Ambienti di Lavoro (SPSAL), Local Health Unit of Reggio Emilia, I-42122 Reggio Emilia, Italy; 2 Occupational Medicine Unit, Direzione Sanità, Italian Railways’ Infrastructure Division, RFI SpA, I-00161 Rome, Italy; dott.pietro.ferraro@gmail.com; 3 AUSL–IRCCS di Reggio Emilia, Laboratorio Analisi Chimico Cliniche e Microbiologiche, Ospedale Civile di Guastalla, I-42016 Guastalla, Italy; simona.peruzzi@ausl.re.it; 4 Department of Medicine and Surgery, University of Parma, I-43126 Parma, Italy; alessandro.zaniboni@unipr.it (A.Z.); silvia.ranzieri@unipr.it (S.R.)

**Keywords:** infants, immunization, physician perception, RSV disease

## Abstract

Respiratory syncytial virus (RSV) is a lead cause of morbidity and hospitalizations in infants. RSV vaccines are currently under development, and preventive options are limited to monoclonal antibodies (mAb). We assessed the knowledge, attitudes and practices for RSV in a sample of general practitioners (GPs) from north-eastern Italy (2021), focusing on the risk perception for infants (age < 8 years) and its potential effectors. We administered an internet survey to 543 GPs, with a response rate of 28.9%. Knowledge status was unsatisfactory, with substantial knowledge gaps found on the epidemiology of RSV and its prevention through mAb. The main effectors of risk perception were identified as having a background in pediatrics (adjusted odds ratio (aOR): 55.398 and 95% confidence interval (95% CI): 6.796–451.604), being favorable towards RSV vaccines when available (aOR: 4.728, 95% CI: 1.999–11.187), while having previously managed an RSV case (aOR: 0.114, 95% CI: 0.024–0.552) and previously recommended hospitalization for cases (aOR: 0.240, 95% CI: 0.066–0.869) were identified as negative effectors. In summary, the significant extent of knowledge gaps and the erratic risk perception, associated with the increasing occurrence in RSV infections, collectively stress the importance of appropriate information campaigns among primary care providers.

## 1. Introduction

Human respiratory syncytial virus (RSV) (genus orthopneumovirus) is a highly contagious viral pathogen belonging to the family of *Pneumoviridae* [1,2,3,4,5,6,7]. Since its original description in 1956 [8], human RSV has emerged as a leading cause of lower respiratory tract infections (LRTI) in children in the first year of age, with a well-defined seasonal trend [3,7]. Moreover, RSV represents a main etiology for severe respiratory infections in older individuals, with high rates of hospitalization, particularly in elderly [9,10].

To date, neither etiologic nor preventive treatments are available. On the one hand, the only available therapeutic option is represented by supportive care (i.e., respiratory support and management of volume depletion) [6,11]. On the other hand, RSV vaccines are commercially unavailable [1,3,9,12,13], and preventive interventions are limited to monoclonal antibodies (mAb) [14,15,16]. Despite their efficacy in avoiding hospitalizations and long-term sequelae, their use is forcibly limited to some high-risk groups (i.e., prematurely-born infants under 6 months of age, and children with certain comorbidities under 2 years of age during the RSV season) [9,17,18,19,20,21].

RSV and SARS-CoV-2, the causative agent of COVID-19, are distinctive vital pathogens [1,9,22], but share several characteristics: improved environmental survival associated with low temperatures and high relative humidity, a high share of mild infections, a substantial unpredictability of the clinical course and the spreading through respiratory inoculation of the upper airways with respiratory secretion from infected individuals [9,23]. Unsurprisingly, there is some evidence that non-pharmaceutic interventions (NPI), implemented to cope with early stages of the SARS-CoV-2 pandemic since March 2020, may have impacted the epidemiology of RSV, eliciting a sudden and earlier end to the epidemic season, with substantially no cases detected in the following months [24,25,26,27,28]. After the lifting of NPI, international data consistently point towards a substantial resurgence of RSV infections. For instance, the 2020–2021 reporting season was characterized by an unprecedented peak in new infections, with a rapid increase in hospital admissions due to bronchiolitis, particularly during the European winter months [29,30,31].

While the SARS-CoV-2 pandemic appears to be far from over, physical distancing and NPI may be required to cope with new waves of infections, and such interventions may in turn unwillingly fuel new waves of RSV [25,31]. In such a setting, not only pediatricians, but also general practitioners (GPs), may be involved in the management of RSV cases. As physicians’ recommendations are critical in modeling the acceptance of clinical options and preventive practices [32], we specifically investigated a sample of Italian GPs on their understanding of RSV disease, their practices regarding the management of RSV disease, and their acceptance of a potential RSV vaccine. Understanding physicians’ knowledge, attitudes and beliefs on RSV and potential RSV vaccines may be useful for targeting specific informative and educative campaigns dedicated to GP that could, in turn, improve the general implementation of available treatment options and effective vaccines, when made available [33,34].

## 2. Materials and Methods

### 2.1. Study Design

A cross-sectional questionnaire-based study was performed between 1 December 2021 and 15 December 2021. A convenience sample of GPs from various Regions of Italy was collected among the participants to a closed mailing list (in total, 543 members). Principal investigators shared a letter via mail outlining the purpose, risks and potential benefits of the study along with a link to the online questionnaire with all participating professionals (Google Forms; Google LLC; Menlo Park, CA, USA). The survey was anonymous, and no personal data such as name, IP address or email address were requested, saved or tracked. No monetary or other compensation was offered to the participants.

### 2.2. Questionnaire

The questionnaire was formulated in Italian through an extensive review of the available literature on RSV [1,2,3,19,33,35,36,37,38,39,40,41,42]. Reliability of the questionnaire was preventively assessed through a test–retest approach, with 10 GPs completing the questionnaire at two different points in time. The two sets of responses were compared through the calculation of a correlation coefficient, and only items with a coefficient >0.80 were included in the final questionnaire. The testing questionnaires were ultimately excluded from the final analyses. All questions were self-reported, and not externally validated (the Authors’ translation of the questionnaire is available as Table A1).

The final questionnaire included the following sections:

1. Individual characteristics of the participants. Characteristics included age, sex, seniority and with or without a background in pediatrics (i.e., having received any post-degree formation and/or training in pediatrics). Moreover, participants were asked to report any previous interaction with RSV; more precisely, whether they had managed any RSV case in their daily practice, diagnosed an RSV infection, required a hospitalization for RSV complicated by LRTI or required shots of mAb for RSV immunoprophylaxis. All the aforementioned iterations were assessed as dichotomous items (ever vs. never).

2. Knowledge Test. A set of 19 true–false statements, and 6 multiple-choice questions covering typical misconceptions on RSV infection (e.g., “In most cases, infants acquire RSV infections from their parents”; false). Internal consistency of the knowledge test was estimated through calculation of Cronbach’s alpha, a statistic calculated from the pairwise correlations between items. A similar knowledge test had previously been validated in order to assess the degree of misconceptions in various settings, and more specifically when dealing with KAP of healthcare providers on infectious diseases and vaccination intentions [32,43,44,45,46,47,48]. A sum score (general knowledge score; GKS) was then calculated as follows: when the participants answered correctly, +1 was scored. On the contrary, a wrong answer or a missing/“don’t know” answer added 0 to the cumulative score. GKS was then dichotomized by median value in higher vs. lower knowledge status.

3. Risk perception. According to Yates, perceived risk may be defined as a function of the perceived probability of an event and its expected consequences [49]. As a consequence, participants were asked to rate the perceived severity (C) and the perceived frequency (F) of RSV infections. As the consequences of RSV infections in infants (age 0 to 8 years) may be strikingly different from those reported among adults (age 18 to 64 years) and the elderly (age 65 and more), C and F were distinctively assessed in the aforementioned age groups through a fully labeled 5-point Likert scale (range: from “not significant”, 1, to “very significant”, 5). Three synthetic risk perception scores (RPS) were then calculated for infants, adults and elders as a product of C and F (i.e., RPS = C × F)

4. Attitudes towards a potential RSV vaccine. Attitudes may be defined as a set of emotions, beliefs and behaviors toward a particular object, person, thing, or event. For the specific aims of this survey, participants were questioned about their willingness to recommend a potential RSV vaccine when available. The following aspects of candidate vaccines were then rated: capability to elicit a mucosal immunity that limits the spreading of the infection, capability to avoid severe infections and LRTI, effectiveness also in the elderly (i.e., individuals ≥ 65 year of age). All aforementioned items were presented as a 5-point fully labeled Likert scale ranging from “totally disagree” (1) to “totally agree” (5), and resulting scores were dichotomized as “somewhat agreeing” (i.e., agree to totally agree) vs. “somewhat disagreeing” (i.e., totally disagree to neutral).

### 2.3. Ethical Considerations

The questionnaire was collected only from subjects who had expressed consent for study participation. Before giving their consent to the survey, participants were briefed regarding the gathered raw data, and that it would be handled anonymously and confidentially and retained only for the time required for analysis. As individual participants cannot be identified through retrieved information, the present study caused no plausible harm or stigma to the respondents. Through its anonymous, observational design and the lack of clinical data, the present study did not configure itself as a clinical trial. According to the Italian law (Gazzetta Ufficiale no. 76, Dated 31 March 2008), a preliminary evaluation by an Ethical Committee was therefore not statutorily required.

### 2.4. Data Analysis

First, synthetic scores (i.e., GSK, RPS for infants, adults and the elderly) were reported as percent values in order to ease their comparison, and then dichotomized by their median in high vs. low estimates.

All continuous variables were then tested for normal distribution through the D’Agostino–Pearson omnibus normality test. Gaussian distribution was rejected for *p* values < 0.10, and variables were then compared through Mann–Whitney or Kruskal–Wallis tests for multiple independent samples. On the other hand, variables passing the normality check (i.e., D’Agostino–Pearson *p* value ≥ 0.10) were compared using the Student’s *t*-test or ANOVA, where appropriate. Association between continuous variables was similarly assessed through calculation of the Pearson’s correlation coefficient or Spearman’s rank correlation coefficient, for variables passing or not passing the normality test.

Categorical variables were reported as percent values, and their distribution in regard to the outcome variable of reporting high concern (i.e., RPS > median value) for RSV in infants was initially analyzed through the chi-squared test. All variables associated with higher concern with *p* value < 0.05 were included in a stepwise binary logistic regression analysis model with calculation of corresponding adjusted odds ratios (aOR), and respective 95% confidence intervals (95% CI). All statistical analyses were performed by means of IBM SPSS Statistics 26.0 for Macintosh (IBM Corp. Armonk, NY, USA).

## 3. Results

### 3.1. Descriptive Analysis: General Characteristics of the Sample

As shown in Table 1, a total of 157 participants eventually completed the online questionnaire (28.9% of the targeted population). Among the respondents, 35 (i.e., 22.3%) were aged 50 years or more (mean age: 43.2 years ± 10.7), and the majority of them were females (60.5%). Overall, the majority of participants (63.1%) had at least 10 years’ seniority as GPs, and only 7.0% acknowledged any background in pediatric settings. Respondents also reported a median of 58 patients in pediatric age group (range 21 to 70; i.e., around 3% to 5% of assisted individuals), with one to six (median, two) visits per week.

### 3.2. Previous Interactions with RSV

Overall, 28.7% of respondents had reportedly managed at least one RSV case in their practice, while 17.8% had diagnosed at least one RSV case and/or recommended the hospitalization because of RSV-associated LRTI. Eventually, 5.1% of respondents had recommended the immunoprophylaxis with mAb.

### 3.3. General Knowledge Test

**Figure 1:**
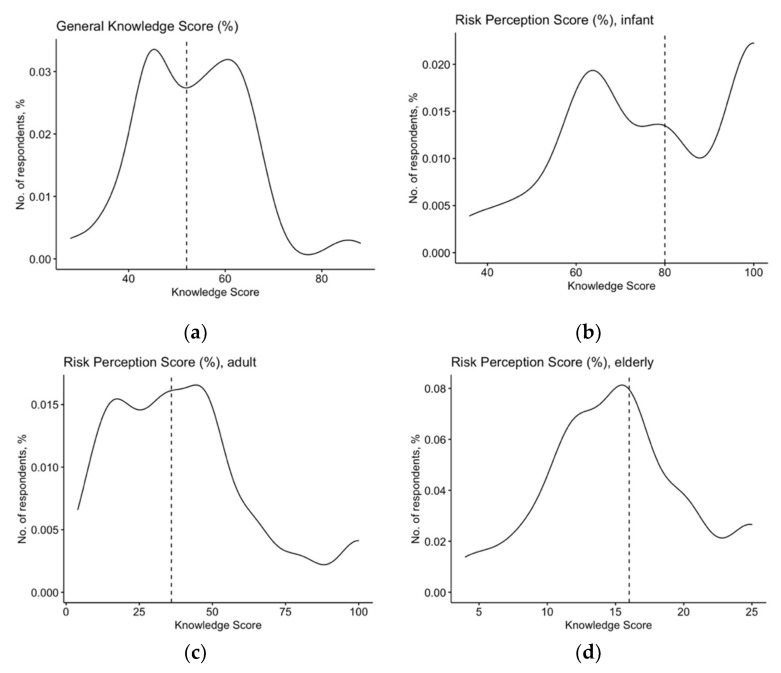
Density plot for general knowledge score (**a**) and risk perception score in infants (**b**), adults (**c**), and the elderly (**d**) in 157 Italian general practitioners participating into the survey. Cumulative scores were substantially skewed for GKS (D’Agostino–Pearson’s normality test: *p* = 0.054), and for RPS in infants (*p* = 0.125, but also visual inspection) and adults (*p* < 0.001) but not for the elderly (*p* = 0.572).

After percent normalization, the mean GKS was unsatisfactory (53.4% ± 11.3; median 52.2%), and its distribution extensively skewed (D’Agostino–Pearson normality test, *p* = 0.054) (Figure 1a). Nevertheless, the internal consistency coefficient amounted to Cronbach’s alpha = 0.746, suggesting an acceptable reliability of the questionnaire.

The detailed results of the knowledge test are reported in Table 2. Briefly, the main uncertainties were associated with the actual epidemiology of RSV infection: even though the large majority of participants had knowledge of the ongoing epidemic of RSV, and the majority of them properly identified the timeframe November–March as the RSV season (61.8%), only 21.7% of respondents correctly associated the majority of RSV-related deaths with the elderly, and 38.9% of participants understood RSV infection as not limited to infants and children. Moreover, most respondents improperly associated RSV-related hospitalizations in terms of raw numbers with pre-term infants and children with known cardiac malformations or respiratory diseases (correct answer, 19.7% and 29.9%, respectively). Actual estimates for incident RSV-related LRTI (around 60% of all LRTI in infants), hospitalization rates (0.5 per 100 infants in their first year of age) and deaths in infants were correctly reported by less than half of the participants (34.4%, 20.4% and 45.2%, respectively). Some clinical aspects of RSV infections were properly shared by respondent physicians (e.g., the higher likelihood of complications when RSV infections are compared to the seasonal influenza virus, 88.5%; the uncomplicated outcome in the large majority of incident cases, 87.9%; the high rate of neurological complications after LRTI, 74.5%; and the role of RSV in eliciting adult asthma, 84.7%), while the lack of specific signs and symptoms was acknowledged by less than half of the participants (correct answers, 45.9%). Although the unavailability of effective vaccines was a shared understanding (81.5%), the significance of mAb in the prophylaxis of LRTI was affected by some knowledge gaps. More precisely, 41.4% of GPs knew that mAb could be used only in preventive settings, 35.7% that commercially available mAb must be delivered every month, during the RSV season, and only one third (i.e., 33.8%) correctly associated the use of mAb in immunoprophylaxis for RSV in pre-term infants. Interestingly, the main knowledge gap affected the role of maternal antibodies, as no more than 7.6% of respondents reportedly understood that protection against RSV would not last for more than 4 months after birth.

### 3.4. Risk Perception

The large majority of respondents characterized RSV as a common disease in infants (87.9%) and the elderly (59.9%), while only 42.7% of them acknowledged its diffusion among adult patients. Similarly, the potential severity of RSV infection was extensively associated with infants (89.8%) and also with the elderly (79.0%), but substantially overlooked for adults (34.4%). The corresponding RPS ranged from 77.6% ± 20.0 for infants (D’Agostino–Pearson *p* = 0.125; Figure 1b), 60.5% ± 21.5 in the elderly (D’Agostino–Pearson *p* = 0.572, Figure 1d) to 40.3% ± 24.8 for adults (D’Agostino–Pearson *p* < 0.001, Figure 1c). Median values were 80.0%, 64.0%, and 48.0%, respectively.

### 3.5. Attitudes towards RSV Vaccine

Overall, 144 out of 157 participants exhibited some degree of acceptance of a potential RSV vaccine when made available (91.7%). When focusing on the design of the potential vaccine, the majority of respondents identified a significant/very significant aspect to be the capability of the vaccine in avoiding complications (98.1%), followed by eliciting mucosal immunity in order to avoid natural infection (89.8%), while only 66.2% stressed the efficiency in individuals aged 65 years or more.

### 3.6. Univariate Analysis

First, no substantial differences were identified in GKS between respondents who had previous professional experience with RSV cases (55.6% ± 11.3 vs. 52.4% ± 11.2; *p* = 0.111) (Table A2), in their understanding (55.6% ± 9.4) compared to those without an occupational background in pediatrics (53.2% ± 11.4, *p* = 0.425) (Table A3). On the other hand, individuals favorable towards the implementation of RSV vaccines (when commercially available) achieved a substantially lower score than those against this option (52.4% ± 10.8 vs. 63.7% ± 11.7; *p* < 0.001) (Table A4).

No substantial correlation was found between GKS and RPS, for infants (rho = −0.140, *p* = 0.081), adults (rho = −0.005, *p* = 0.952) and the elderly (rho = −0.122, *p* = 0.127). On the contrary, RPS scores were correlated with each other (Table 3), i.e., a higher level of concern for an age group was correlated with a higher concern for other groups, and vice versa.

**Figure 2:**
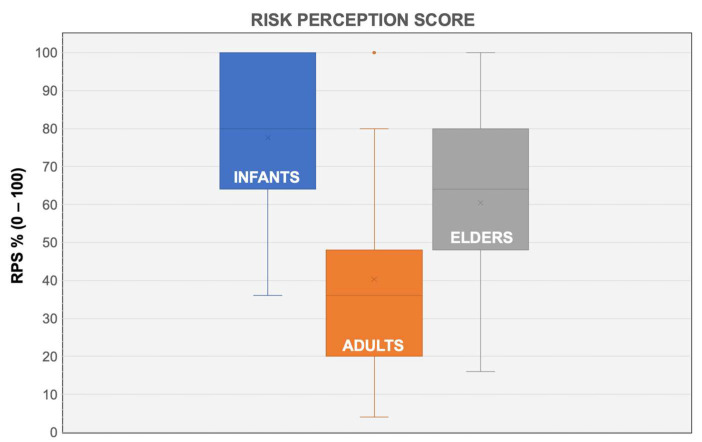
Box and whiskers plot with the comparisons of RSP for infants, adults and elders. The score was substantially greater for infants (77.6% ± 20.0) compared to both adults (40.3% ± 24.8, *p* < 0.001) and elders (60.5% ± 21.5, *p* < 0.001).

A comparison for RPS in infants, adults and elders identified a significantly higher RPS compared to both elders and adults (for both comparisons, *p* < 0.001). In turn, elders were associated with a RPS being significantly greater than that reported for adults (see Figure 2).

In the univariate analysis for dichotomous variables (Table 4), a higher risk of concern was associated with having any background in pediatrics (14.0% vs. 3.0%, *p* = 0.023), and exhibiting a favorable attitude towards a potential RSV vaccine (100% vs. 87.0%, *p* = 0.011). On the contrary, a higher concern for RSV infections in infants was negatively associated with the male gender (22.8% vs. 49.0%, *p* = 0.002), having previously managed any RSV case (17.5% vs. 35.0%, *p* = 0.032) and having previously recommended hospitalization for LRTI following RSV infections (7.0% vs. 24.0%, *p* = 0.014).

### 3.7. Regression Analysis

In regression analysis, higher risk perception for RSV (Table 4) was assessed through a model that included the following explanatory variables (all of them associated with *p* < 0.05 at univariate analysis): male gender, having a background in pediatrics, having previously managed an RSV case, having previously recommended hospitalization for LRTI infections associated with RSV, and being favorable/highly favorable towards an RSV vaccine, when made available.

Eventually, having a background in pediatrics (aOR: 55.398, 95% CI: 6.796 to 451.604) and being somewhat favorable towards a potential RSV vaccine (aOR: 4.728, 95% CI: 1.999 to 11.187) were identified as the main positive effectors for having some degree of concern for RSV infections in infants. On the contrary, previous experiences with RSV infections, including having managed a case (aOR: 0.114, 95% CI: 0.024 to 0.552) and having previously recommended the hospitalization for RSV-related LRTI infections (aOR: 0.240, 95% CI: 0.066 to 0.869) were characterized as main negative effectors.

## 4. Discussion

Human RSV is associated with a substantial burden of disease in children and elders [7,10,35,38,42,50,51]. NPI, due to the SARS-CoV-2 pandemic, have led to the substantial disappearance of the conventional seasonal trend for RSV infections [1,24,29,30,31,52,53,54,55,56,57], that have been in turn followed by an unprecedented surge in incident cases [29,30,31,52,58,59]. Accurate statistics are unavailable, but GPs and pediatricians usually manage up to 97% of yearly incident cases as outpatients [16,60,61], representing a substantial burden for primary practitioners still struggling with the requirements of the SARS-CoV-2 pandemic.

Our survey found the understanding of RSV to be unsatisfactory and the risk perception to be erratic, with substantial underscoring of RSV infections among the elderly. A reasonable explanation may be found in the knowledge gaps of participants, as the occurrence of RSV infections and their potential consequences in the elderly were correctly acknowledged by a reduced share of respondents (21.7% and 38.9%, respectively). The substantial misbeliefs of healthcare providers, particularly on RSV in older individuals is consistent with several previous studies [62,63,64].

However, some conflicting results were identified also regarding RSV infections of infants and children. On the one hand, a background in pediatrics was a strong predictor for higher risk perception (aOR: 55.398, 95% CI: 6.796 to 451.604), while having previously managed an RSV case (aOR: 0.114, 95% CI: 0.024 to 0.552) and having previously recommended hospitalization for RSV cases (aOR: 4.728, 95% CI: 1.999 to 11.187) were characterized as negative predictors. In other words, respondents having had some actual experiences with RSV cases underscored the potential consequences of the infection in pediatric age. Even a direct comparison of cumulative score did not identify any substantial difference in RPS between professionals claiming any experience in RSV management or not (Table A2; 74.5% ± 18.0 vs. 78.8% ± 20.7, *p* = 0.195).

This finding was somewhat unexpected, as personal experiences usually represent a strong predictor for later behaviors [65,66,67], but previous reports on RSV-associated attitudes and beliefs have stressed an extensive underestimation of potential occurrence and health consequences of RSV infections. For example, in the study by Wilcox et al. [63], including a total of 37 obstetrics and 151 midwives, the majority of respondents acknowledged RSV infections as moderately common (61.7%) and severe (73.4%), with similar estimates for bronchiolitis (66.0% for frequency, and 72.3% for severity). Similarly, in a study on 543 infectious disease specialists from North America, among 293 professionals managing acute respiratory illnesses in adults, around 34.8% did not report either ordering or recommending RSV testing [64]. A possible explanation may be found in the characteristics of this convenience sample and their experience with potentially dismal outcomes of RSV infections. While RSV is potentially associated with severe consequences in infants, irrespective of their health status [11,68,69], the majority of annual deaths occur in the elderly [10,12,41,42,70,71,72], but the causal agent remains undiagnosed in the majority of cases [10,12,40]. As most of the participants actually lacked any background in pediatrics, having only a limited daily practice in the management of pediatric infectious diseases, the scarce familiarity with the actual severity of this pathogen may have led them to improperly underscore the specific health risks. In fact (Table A3), professionals with a background in pediatrics did not show an increased understanding in RSV (GKS 55.6% ± 9.4 vs. 53.2% ± 11.4, *p* = 0.425) compared to those without, but they scored a substantially greater risk perception in infants (94.5% ± 9.3 vs. 76.3% ± 20.0, *p* < 0.001), and lower for adults (19.6% ± 6.1 vs. 41.8% ± 24.0, *p* < 0.001) and elders (39.3% ± 18.4 vs. 62.1% ± 20.9, *p* = 0.002).

Moreover, being favorable towards RSV vaccines was a strong predictor for higher risk perception in infants (aOR 4.728; 95% CI 1.999 to 11.187), a result that was somewhat consistent with previous reports on healthcare providers [63]. Despite significant shortcomings (i.e., the potential induction of severe side effects such as the enhanced RSV disease, an aggressive immune response sustained by CD4+ T cells; the potential lack of efficacy in pre-term infants), effective vaccines represent promising opportunities to achieve a better management of RSV infections in all age groups [1,73,74]. Unfortunately, vaccines for RSV still remain commercially unavailable [73,75]. Notwithstanding, the large majority of respondents exhibited substantial support for their implementation, when possible (i.e., 91.7% of all participants).

All participants reporting the previous management of RSV cases (No. = 45) were somewhat favorable towards the implementation of RSV vaccines (Table A5), as well as for the respondents with any background in pediatrics (No. = 11). Despite conflicting results from previous studies on RSV [62,63,64], such results would therefore reinstate the potential role for personal experiences in the modeling of attitudes and behaviors [65,66,67]. Interestingly, higher RPS in adults and elders were unrelated with the acceptance or RSV vaccines and being favorable or not towards RSV vaccines did not result in significant differences for RPS in adults (40.4% ± 24.9 vs. 38.8% ± 5.3, *p* = 0.529) and elders (60.5% ± 22.1 vs. 60.3% ± 13.3, *p* = 0.969; Table A4). In other words, participating GPs not only underestimated the potential risk associated with RSV but did not seem to identify RSV vaccines as a potential strategy to cope with the burden of disease represented by this pathogen in adults. However, the present sample characterized the potential vaccination of individuals aged ≥ 65 years as a potential priority (66.2%), and this was consistent with a previous study from the United States, where primary care providers characterized RSV as an important pathogen in older individuals, and for those with cardiopulmonary disease and immuno-compromising conditions [33].

There were some limits to this study. To our knowledge, there is a substantial lack of KAP studies on RSV and primary healthcare providers from European settings. Despite its novelty and potential significance, our study is affected by several shortcomings. First, internet-based surveys are affected by the substantial “self-selection” of participants [43,76,77], that in turn can lead to the substantial oversampling of certain sub-groups within the targeted population (e.g., subjects familiar with sharing personal information through internet and social media, individuals exhibiting a proactive attitude or greater knowledge about the assessed topic, etc.). Similarly, not participating could be understood as a negative attitude or a lack of knowledge about the targeted topic [76]. The potential self-selection of participants may have been somewhat moderated through a sampling strategy that prioritized a homogenous subgroup of medical professionals (GP) participating in a common discussion group. Despite the preventive selection by a group manager, as the answers from participating professionals were not externally validated, we cannot rule out that some of the respondents did not fully adhere to our selection criteria, and this would further compromise the actual representation of the sample.

Second, the potential generalizability of our results was compromised by the small sample size. Assuming the level of concern towards RSV reported in the aforementioned study by Hurley et al. [33] as a reference (i.e., 37% of adults without any previous cardiopulmonary disease and 71% for elders), an error of 5% (0.05) and a power of 95%, minimum sample size should have been between 316 and 358. As a consequence, our study should be acknowledged as an exploratory one, needing further and more extensive research, particularly for occupational groups involved in the management of children and newborns (i.e., pediatricians, midwives and obstetricians) [63,64].

Third, social desirability bias may have substantially affected the knowledge test. In studies on attitudes and beliefs, participants often report “common sense” answers or those perceived as more “appropriate” to fit with the aim of the questionnaire, that are therefore prioritized over the actual understanding of the focused theme [32,78,79,80]. As a topic such as RSV is reasonably affected by significant knowledge gaps not only in the general population [62], but also in caregivers without a specific pediatric background [33,63,64], the consequences of this shortcoming are potentially substantial. Therefore, we cannot rule out that our results could have also ultimately overstated the share of individuals having an effective understanding of RSV.

## 5. Conclusions

In conclusion, RSV is a common infection in infants, and a substantial cause of morbidity and mortality even in older individuals. Therefore, it represents a clinical problem for all primary care providers, including GPs. In this convenience sample of Italian GPs, participants exhibited an unsatisfactory understanding of RSV and risk factors as well as erratic RPS, particularly for older individuals. When dealing with RSV infections in infants, it is reasonable that personal experiences may have led to a better understanding of RSV and the actual burden. While high-income countries are facing unprecedented epidemics of RSV infections in infants and newborns, increasing evidence leads to acknowledging RSV as a significant cause of morbidity and mortality in older individuals. Innovative and more specifically tailored formation of primary care providers are therefore required in order to increase their capability to cope with the needs of their patients.

## Figures and Tables

**Table 1:** Characteristics of the 157 Italian general practitioners (GPs) participating in the survey on knowledge, attitudes and practices for respiratory syncytial virus (Note: SD = Standard Deviation).

Variable	No./157%	Average ± SD
Gender		
Male	62, 39.5%	
Female	79, 60.5%	
Age (years)		43.2 ± 10.7
Age ≥ 50 years	35, 22.3%	
Seniority as GP		16.9 ± 10.9
Seniority ≥ 10 years	99, 63.1%	
Any occupational background in Pediatrics	11, 7.0%	
Previously managed RSV cases	45, 28.7%	
Previously diagnosed RSV cases	28, 17.8%	
Previously required hospitalization for RSV	28, 17.8%	
Previously required mAb immunoprophylaxis for RSV	8, 5.1%	
Acknowledging RSV infection as frequent/very frequent in		
infants	138, 87.9%	
adults	67, 42.7%	
elderly	94, 59.9%	
Acknowledging RSV infection as severe/very severe in…		
infants	141, 89.8%	
adults	54, 34.4%	
elderly	124, 79.0%	
General Knowledge Score (%)		53.4 ± 11.3
General Knowledge Score > median (52.0%)	76, 48.4%	
Risk Perception Score for infants		77.6 ± 20.0
Risk Perception Score for infants > median (80.0%)	57, 36.3%	
Risk Perception Score for adults		40.3 ± 24.8
Risk Perception Score for adults > median (48.0%)	68, 43.3%	
Risk Perception Score for elderly		60.5 ± 21.5
Risk Perception Score for elderly > median (64.0%)	41, 26.1%	
Favorable/Highly favorable towards an RSV vaccination when made available	144, 91.7%	
Acknowledging as significant/very significant aspects for candidate RSV vaccines		
Avoiding natural infection (i.e., mucosal immunity)	141, 89.8%	
Avoiding complications (i.e., LRTI)	154, 98.1%	
Being efficient also in individuals aged 65 years or more	104, 66.2%	

**Table 2:** Knowledge test showing response distribution of presented items proposed to the 157 medical professionals participating into the survey on respiratory syncytial virus (RSV) and contributing to the assessment of general knowledge score (GKS) (Cronbach’s alpha = 0.746).

Statement	Correct Answer	Total (No./157)
Nearly all RSV infections occur in infants and children.	False	61, 38.9%
In most cases, infants acquire RSV infections from their parents.	False	88, 56.1%
In most cases, RSV evolves in an uncomplicated influenza-like illness.	True	138, 87.9%
Lower respiratory tract infections from RSV is deprived of specific signs/symptoms.	True	72, 45.9%
In Europe, RSV season spans from:		
November–March	True	97, 61.8%
October–February	False	47, 29.9%
September–January	False	13, 8.3%
SARS-CoV-2 and RSV have the same means of transmission.	True	157, 100%
Safe and effective vaccines against RSV are commercially available.	False	128, 81.5%
Monoclonal antibodies can be used against RSV only as immunoprophylaxis.	True	65, 41.4%
Immunoprophylaxis for RSV should be delivered:		
Every two months, during RSV season	False	28, 17.8%
Every month, during RSV season	True	56, 35.7%
Only at the beginning of RSV season	False	73, 46.5%
Globally, RSV causes a total … deaths in children < 1 age:		
43,800	True	71, 45.2%
430,800	False	73, 46.5%
Around 1,000,000	False	13, 8.3%
According to available figures, RSV causes every year a total of … hospitalizations:		
2 million	True	62, 39.5%
10 million	False	75, 47.8%
22 million	False	16, 10.2%
Do not know	-	4, 2.5%
According to WHO estimated, RSV causes … of lower respiratory tract infections:		
40%	False	92, 58.6%
60%	True	54, 34.4%
75%	False	11, 7.0%
RSV infections may cause severe neurological complications.	True	117, 74.5%
RSV has been acknowledged as a risk factor for adult asthma.	True	133, 84.7%
Seroprevalence for RSV reaches 100% before 2nd year of age.	True	84, 53.5%
Maternal antibodies reduce the risk of RSV infections during first 4 months of age.	False	12, 7.6%
Hospitalization rate for RSV during the first year of age may reach:		
0.5 per 100	True	32, 20.4%
1 per 100	False	53, 33.8%
5 per 100	False	72, 45.9%
The majority of patients hospitalized for RSV are affected by chronic respiratory disorders and cardiac malformations.	False	47, 29.9%
The majority of hospitalizations for RSV occur among pre-term infants.	False	31, 19.7%
According to available recommendations, mAb should be used only in preterm infants.	True	53, 33.8%
Around three quarters of all RSV-related deaths occurs in subjects older than 65 years.	True	34, 21.7%
During SARS-CoV-2 pandemic, global incidence of RSV infections has decreased.	True	111, 70.7%
To date (December 2021), Italy is affected by an RSV epidemic.	True	132, 84.1%
RSV natural infection elicit a long-lasting immunity.	False	86, 54.8%
Severe complications are more likely in RSV than in seasonal influenza infections.	True	139, 88.5%

**Table 3:** Correlation between synthetic scores, i.e., general knowledge score (GKS) and risk perception score (RPS), calculated for infants, adults and the elderly. Spearman’s correlation test (rho) with their respective *p* value.

Variable	GKS	RPS for Infants	RPS for Adults	RPS for Elders
GKS	-	−0.140 (*p* = 0.081)	−0.005 (*p* = 0.952)	−0.122 (*p* = 0.127)
RPS for infants	−0.140 (*p* = 0.081)	-	−0.194 (*p* = 0.015)	−0.168 (*p* = 0.036)
RPS for adults	−0.005 (*p* = 0.952)	−0.194 (*p* = 0.015)	-	−0.610 (*p* < 0.001)
RPS for elders	−0.122 (*p* = 0.127)	−0.168 (*p* = 0.036)	−0.610 (*p* < 0.001)	-

**Table 4:** Univariate analysis of factors associated with higher risk perception for RSV (i.e., risk perception score > median) compared to low risk perception (i.e., risk perception score ≤ median) in infants and elderly. Comparisons were performed by means of chi-squared test (with Yates’ correction). Adjusted odds ratios (aOR) and their respective 95% confidence intervals were calculated through binary logistic regression analysis, including as explanatory variables, all factors associated in univariate analysis with the outcome variables with *p* < 0.05.

Variable	Risk Perception for Infants			
	High Concern No./57%	Low Concern No./100%	*p* Value	aOR (95% CI)
Male gender	13, 22.8%	49, 49.0%	0.002	0.472 (0.201; 1.107)
Age > 50 years	9, 15.8%	26, 26.0%	0.201	-
Seniority ≥ 10 years	37, 64.9%	62, 62.0%	0.848	-
GKS > median (52.0%)	22, 38.6%	54, 54.0%	0.091	-
RPS, adults > median (48.0%)	22, 38.6%	46, 46.0%	0.464	-
RPS, elders > median (64.0%)	10, 17.5%	31, 31.0%	0.098	-
Any background in pediatrics	8, 14.0%	3, 3.0%	0.023	55.398 (6.796; 451.604)
Previously managed any RSV case	10, 17.5%	35, 35.0%	0.032	0.114 (0.024; 0.552)
Previously diagnosed any RSV case	12, 21.1%	16, 16.0%	0.563	-
Previously recommended hospitalization for RSV infection	4, 7.0%	24, 24.0%	0.014	0.240 (0.066; 0.869)
Previously recommended mAb	4, 7.0%	4, 4.0%	0.653	-
Favorable/Highly favorable towards RSV vaccine	57, 100%	87, 87.0%	0.011	4.728 (1.999; 11.187)

Notes: aOR = adjusted odds ratio (i.e., odds ratio calculated through binary logistic regression); 95% CI = 95% confidence interval.

## Data Availability

The data presented in this study are available on request from the corresponding author.

## Appendix A

**Table A1:** Authors’ translation of the items included in the questionnaire.

**Section 1. Your personal experience with RSV infections:** **during your clinical practice.**	
Have you previously managed any RSV case?	[yes] [no] [no answer]
Have you previously diagnosed any RSV case?	[yes] [no] [no answer]
Have previously required any hospitalization for RSV?	[yes] [no] [no answer]
Have you previously required mAb immunoprophylaxis for RSV?	[yes] [no] [no answer]
**Section 2. At your knowledge (please mark the correct answer)**	
1. Nearly all RSV infections occur in infants and children.	[true] [false] [do not know]
2. In most cases, infants acquire RSV infections from their parents.	[true] [false] [do not know]
3. In most cases, RSV evolves in an uncomplicated influenza-like illness.	[true] [false] [do not know]
4. Lower respiratory tract infections from RSV is deprived of specific signs/symptoms.	[true] [false] [do not know]
5. In Europe, RSV season spans from:	
November–March	[ ]
October–February	[ ]
September–January	[ ]
6. SARS-CoV-2 and RSV have the same means of transmission.	[true] [false] [do not know]
7. Safe and effective vaccines against RSV are commercially available.	[true] [false] [do not know]
8. Monoclonal antibodies can be used against RSV only as immunoprophylaxis.	[true] [false] [do not know]
9. Immunoprophylaxis for RSV should be delivered:	
Every two months, during RSV season	[ ]
Every month, during RSV season	[ ]
Only at the beginning of RSV season.	[ ]
10. Globally, RSV causes a total … deaths in children < 1 age:	
43,800	[ ]
430,800	[ ]
Around 1,000,000	[ ]
11. According to available figures, RSV causes every year a total of … hospitalizations:	
2 million	[ ]
10 million	[ ]
22 million	[ ]
Do not know.	[ ]
12. According to WHO estimated, RSV causes … of lower respiratory tract infections:	
40%	[ ]
60%	[ ]
75%	[ ]
13. RSV infections may cause severe neurological complications.	[true] [false] [do not know]
14. RSV has been acknowledged as a risk factor for adult asthma.	[true] [false] [do not know]
15. Seroprevalence for RSV reaches 100% before 2nd year of age.	[true] [false] [do not know]
16. Maternal antibodies reduce the risk of RSV infections during first 4 months of age.	[true] [false] [do not know]
17. Hospitalization rate for RSV during the first year of age may reach:	
0.5 per 100	[ ]
1 per 100	[ ]
5 per 100	[ ]
18. The majority of patients hospitalized for RSV are affected by chronic respiratory disorders and cardiac malformations.	[true] [false] [do not know]
19. The majority of hospitalizations for RSV occur among pre-term infants.	[true] [false] [do not know]
20. According to available recommendations, mAb should be used only in preterm infants.	[true] [false] [do not know]
21. Around three quarters of all RSV-related deaths occurs in subjects older than 65 years.	[true] [false] [do not know]
22. During SARS-CoV-2 pandemic, global incidence of RSV infections has decreased.	[true] [false] [do not know]
23. To date (December 2021), Italy is affected by a RSV epidemic.	[true] [false] [do not know]
24. RSV natural infection elicit a long-lasting immunity.	[true] [false] [do not know]
25. Severe complications are more likely in RSV than in seasonal influenza infections.	[true] [false] [do not know]
3. Please rate the following items from “not significant” (1) to “very significant” (5)	
How do you perceive the frequency of RSV infections?	
In infants (age 0 to 8 years)	[1] [2] [3] [4] [5]
In adults (age 18 to 64 years)	[1] [2] [3] [4] [5]
In elderly (age ≥ 65 years)	[1] [2] [3] [4] [5]
How do you perceive the severity of RSV infections?	
In infants (age 0 to 8 years)	[1] [2] [3] [4] [5]
In adults (age 18 to 64 years)	[1] [2] [3] [4] [5]
In elderly (age ≥ 65 years)	[1] [2] [3] [4] [5]
4. Are you favorable towards the implementation of a RSV vaccine in the specific vaccine schedule, if commercially available ?(1 = totally disagree; 5 = totally agree)	[1] [2] [3] [4] [5]
5. In the design of a candidate RSV vaccine, which aspects are of specific importance, from your point of view? (1 = totally disagree; 5 = totally agree)	
avoiding natural infection (i.e., mucosal immunity)	[1] [2] [3] [4] [5]
avoiding complications (i.e., LRTI)	[1] [2] [3] [4] [5]
being efficient also in individuals aged 65 years or more.	[1] [2] [3] [4] [5]
6. Please provide some general information about you	
Year of birth:	______________
Year of medical qualification as GP:	______________
You identify yourself as:	[male] [female] [no answer]
Do you have any previous professional experience in Pediatric settings?	[yes] [no] [no answer]
At the moment, how many individuals aged less than 14 years do you assist as GP?	_______________
At the moment, how many medical consultations/visits do you perform by week in individuals aged 14 years or less?	_______________

Notes: the present questionnaire can be shared and modified by the end user. Please cite the present paper.

**Table A2:** Comparison of cumulative scores by having or not previously managed any RSV case (Note: SD = Standard Deviation).

Variable	Previously Managed Any RSV Case (Average ± SD)		*p* Value (Mann–Whitney Test)
	Yes (No. = 45)	No (No. = 112)	
GKS (%)	55.6 ± 11.3	52.4 ± 11.2	0.111
RPS for infants (%)	74.5 ± 18.0	78.8 ± 20.7	0.195
RPS for adults (%)	37.2 ± 30.5	41.5 ± 22.1	0.399
RPS for elders (%)	51.5 ± 22.1	64.0 ± 20.2	0.002

**Table A3:** Comparison of cumulative scores by being or not favorable towards RSV vaccines when available (Note: SD = Standard Deviation).

Variable	Being Favorable towards RSV Vaccines (When Available) (Average ± SD)		*p* Value (Mann Whitney Test)
	Yes (No. = 144)	No (No. = 13)	
GKS (%)	52.4 ± 10.8	63.7 ± 11.7	<0.001
RPS–infants (%)	78.8 ± 20.2	64.0 ± 11.3	<0.001
RPS–adults (%)	40.4 ± 25.9	38.8 ± 5.3	0.529
RPS–elders (%)	60.5 ± 22.1	60.3 ± 13.3	0.969

**Table A4:** Comparison of cumulative scores by having or not any background in pediatrics (Note: SD = Standard Deviation).

**Variable**	**Any Background in Pediatrics** **(Average ± SD)**		***p* Value** **(Mann Whitney Test)**
	**Yes** (**No. = 11**)	**No** (**No. = 146**)	
GKS (%)	55.6 ± 9.4	53.2 ± 11.4	0.425
RPS–infants (%)	94.5 ± 9.3	76.3 ± 20.0	<0.001
RPS–adults (%)	19.6 ± 6.1	41.8 ± 25.0	<0.001
RPS–elders (%)	39.3 ± 18.4	62.1 ± 20.9	<0.001

**Table A5:** Association of being favorable towards RSV vaccines with main variables (univariate analysis, chi-squared test with Yates’ correction).

Variable	Being Favorable towards RSV Vaccine (When Available)		
	Yes No./144, %	No No./13, %	*p* Value
Male Gender	54, 37.5%	8, 61.5%	0.161
Age > 50 years	29, 20.1%	6, 46.2%	0.070
Seniority ≥ 10 years	89, 61.8%	10, 76.9%	0.434
GKS > median (52.0%)	63, 43.8%	13, 100%	<0.001
RPS, infants > median (48.0%)	57, 39.6%	0, -	0.011
RPS, adults > median (48.0%)	65, 45.1%	3, 23.1%	0.213
RPS, elders > median (64.0%)	38, 26.4%	3, 23.1%	1.000
Any background in Pediatrics	11, 7.6%	0, -	0.641
Previously managed any RSV case	45, 31.3%	0, -	0.039
Previously diagnosed any RSV case	28, 19.4%	0, -	0.169
Previously recommended hospitalization for RSV infection	28, 19.4%	0, -	0.169
Previously recommended mAb	8, 5.6%	0, -	0.831
